# Low-Temperature Rheological Performance and Microscopic Aging Mechanism of SBS-Modified Asphalt Under Thermal-Oxidative and UV Aging

**DOI:** 10.3390/ma19163489

**Published:** 2026-08-18

**Authors:** Keyan Ma, Yuwen Shi, Fucheng Guo, Yangyang Guo, Zhengchen Li, Di Wang

**Affiliations:** 1 Gansu Province Transportation Planning Survey & Design Institute Co., Ltd., Lanzhou 730010, China; 2 Key Laboratory of Road & Bridge and Underground Engineering of Gansu Province, Lanzhou Jiaotong University, Lanzhou 730070, China; 3 Department of Civil Engineering, University of Ottawa, 800 King Edward Ave., Ottawa, ON K1N 6N5, Canada

**Keywords:** UV aging, SBS-modified asphalt, low-temperature performance, aging mechanism

## Abstract

Ultraviolet (UV) radiation in high-altitude regions critically accelerates asphalt aging by inducing surface oxidation, molecular chain scission, and loss of low-temperature crack resistance. However, systematic comparisons of the macro-rheological and micro-chemical evolution between base asphalt and SBS-modified asphalt under UV aging remain insufficient. In this study, two types of asphalt (virgin and SBS-modified) were subjected to three aging protocols, namely short-term thermal oxidation (RTFOT), long-term thermal oxidation (PAV), and equivalent UV radiation for 13 h, 26 h, and 37 h. Low-temperature rheological properties were evaluated using the bending beam rheometer (BBR), while atomic force microscopy (AFM) and Fourier transform infrared spectroscopy (FTIR) characterized the microstructural and chemical changes. The results show that long-term thermal oxidation causes the most severe deterioration of low-temperature rheological performance, whereas short-term thermal oxidation and 13 h UV aging exhibit comparable effects. For SBS-modified asphalt, extending UV exposure from 13 h to 37 h leads to progressive stiffening and loss of relaxation capacity at −12 °C and −18 °C. However, the m-value shows a non-monotonic response at −24 °C, indicating that the temperature dependence of UV aging is more complex at extremely low temperature. For base asphalt, aging promotes the formation and subsequent agglomeration of bee-like structures. For SBS-modified asphalt, the sulfoxide index increases monotonically, while the carbonyl index first increases and then decreases. Although 13 h UV aging and RTFOT produce similar macroscopic outcomes, their mechanisms differ fundamentally, where UV aging is hypothesized to act primarily via photon-induced bond scission, whereas thermal oxidation proceeds through radical chain reactions.

## 1. Introduction

Styrene–butadiene–styrene (SBS) polymer-modified asphalt has become one of the most widely used modified binders in pavement engineering owing to its favorable high-temperature rutting resistance, low-temperature cracking resistance and fatigue performance [1]. In contrast, conventional base asphalt often suffers from insufficient flexibility at low temperatures, leading to brittle fracture, and exhibits excessive softening at high temperatures, which compromises rutting resistance. To overcome these limitations, SBS is incorporated as a polymeric modifier. The SBS modifier establishes a three-dimensional polymer network within the asphalt matrix, wherein the polystyrene hard segments form physical crosslinking points while the polybutadiene soft segments adsorb aromatic components to form a continuous elastic network. This multi-scale collaborative structure not only retains sufficient chain relaxation capacity at low temperatures, as manifested in higher creep rates and lower stiffness moduli, but also provides enhanced resistance to thermal-oxidative aging by limiting the diffusion of oxygen molecules into the bulk phase [2]. As a result, SBS-modified asphalt exhibits markedly improved low-temperature cracking resistance and high-temperature stability compared with unmodified base asphalt.

Despite these advantages, SBS-modified asphalt remains susceptible to aging under the coupling effects of thermal oxidation and ultraviolet radiation [3]. Extensive research has been conducted on thermal-oxidative aging using standardized laboratory protocols, namely the rolling thin film oven test (RTFOT) for short-term aging and the pressure aging vessel (PAV) for long-term aging [4,5]. It has been documented that RTFOT increases the viscosity of SBS-modified asphalt and increases its penetration, ductility and softening point, while PAV induces more severe hardening with pronounced reductions in ductility [6]. However, the existing literature predominantly focuses on thermal-oxidative aging, and the aging response of SBS-modified asphalt under prolonged UV irradiation remains comparatively understudied [7]. Given that UV aging operates through fundamentally different mechanisms, thermal-oxidative aging follows free radical chain reactions driven by thermal energy, whereas UV aging is thought to directly cleave chemical bonds via high-energy photons, the degradation pathways and performance deterioration patterns induced by UV exposure cannot be reliably inferred from thermal-oxidative studies alone [8,9,10]. This knowledge gap necessitates dedicated investigation into the UV aging behavior of SBS-modified asphalt [11].

The Hexi Corridor is situated at the northern edge of the Qinghai–Tibet Plateau, adjoining the Qilian Mountains to the south (average elevation larger than 4000 m) and the Inner Mongolia Plateau to the north. The corridor itself has a general elevation of approximately 1000–1500 m, forming a transitional zone between the plateau and the lower plains. This geographical setting, combined with high atmospheric transparency, results in intense solar and UV radiation in the region [12]. Field investigations have indicated that UV radiation in such high-altitude regions can be 2–3 times greater than that in inland plain areas. While the region experiences minimal natural precipitation-induced aging, the combination of significant temperature fluctuations and strong UV radiation imposes severe durability challenges on asphalt pavements. Large temperature differentials readily induce thermal stress accumulation, while intense UV radiation accelerates the oxidative hardening of asphalt binders, leading to embrittlement and premature cracking [13]. In this context, the low-temperature rheological performance of asphalt after prolonged UV exposure becomes critically important [14].

Elucidating the aging mechanisms of SBS-modified asphalt is a prerequisite for the targeted regulation of its material properties. The aging mechanisms of SBS-modified asphalt under different environmental conditions have been characterized using atomic force microscopy (AFM) and Fourier transform infrared spectroscopy (FTIR) [15]. AFM enables the observation of surface “bee-like structures,” whose number, area fraction and roughness evolution serve as sensitive indicators of aging-induced microstructural changes [16]. The morphological characteristics of these bee-structures, including their size and spatial distribution, have been shown to be highly dependent on sample preparation and thermal history [17]. FTIR analysis allows quantitative determination of the carbonyl and sulfoxide indexes, which reflect the degree of oxidative aging. However, most AFM- and FTIR-based studies have focused on thermal-oxidative aging, with limited attention devoted to UV-induced degradation [18]. Moreover, the aging mechanism of SBS-modified asphalt under UV exposure is fundamentally different from that under thermal-oxidative conditions: the SBS polybutadiene double bonds are highly susceptible to UV photon attack, leading to chain scission and degradation of the polymer network, whereas thermal-oxidative aging predominantly induces crosslinking and hardening of the asphalt matrix [19]. These mechanistic differences warrant systematic investigation into the UV aging behavior of SBS-modified asphalt from both rheological and microstructural perspectives [20].

This study employed the equivalent radiation method to simulate the high-altitude UV environment. Three UV aging durations, namely 13 h, 26 h and 37 h, were selected on the basis of the equivalent radiation conversion relationship between laboratory accelerated aging and field service conditions in high-altitude regions. For comparison, short-term thermal-oxidative aging (RTFOT) and long-term thermal-oxidative aging (PAV) were also conducted on the same SBS-modified asphalt. The low-temperature rheological properties of the aged binders were evaluated using the bending beam rheometer (BBR) in terms of creep stiffness modulus (S) and creep rate (m). The selection of appropriate rheological indices is critical for accurately evaluating aged binder performance, as different indices may exhibit varying sensitivity to different aging mechanisms [21]. The microstructural changes induced by different aging modes were characterized by AFM, with emphasis on the evolution of surface topography and bee-like structural parameters, while the chemical aging mechanism was probed by FTIR through quantitative analysis of the carbonyl index and sulfoxide index. This integrated macro–micro investigation aims to elucidate the differential aging response of SBS-modified asphalt under UV and thermal-oxidative conditions, thereby providing a theoretical basis for material selection and performance evaluation in high-UV regions.

## 2. Materials and Methods

### 2.1. Raw Materials

(1) Base asphalt

Zhenhai 90# base asphalt is selected as the comparison sample and modified base material. The base asphalt was provided by Gansu Province Transportation Planning Survey & Design Institute Co., Ltd. (Lanzhou, China). The technical indicators of the base asphalt binder were measured as shown in Table 1, meeting the specification requirement (JTG F40-2023) [22].

(2) SBS-modified asphalt

SBS-modified asphalt was prepared by adding a linear SBS modifier into base asphalt. In this study, an SBS content of 3.7% was used. The SBS-modified asphalt was prepared in the laboratory using a high-shear mixer. The base asphalt (Zhenhai 90#) was first heated to 175–180 °C until fully fluid. The SBS modifier (linear type, styrene/butadiene ratio = 30/70, Mn =100,000–150,000 g/mol, supplied by Survey & Design Institute Co., Ltd. (Lanzhou, China.) at a dosage of 3.7 wt% by weight of base asphalt was then added gradually under low-speed stirring (nearly 500 rpm) and allowed to swell for 30 min at 175–180 °C. The mixture was subsequently sheared at 3000–4000 rpm for 60 min at the same temperature using a high-shear mixer. After shearing, the blend was transferred to an oven and developed at 163 °C for 60 min under gentle stirring to ensure homogeneity. The entire preparation process was conducted in open air without the addition of chemical stabilizers. The prepared SBS-modified asphalt was stored in sealed metal containers at room temperature and reheated to 135–140 °C with gentle stirring prior to testing. The SBS content of 3.7% used in this study has been widely verified by previous publications and engineering experience for its high cost-effectiveness. All of the indexes meet the requirements of the specification.

### 2.2. Aging Methods

#### 2.2.1. Short-Term Thermal Oxygen Aging Test

The RTFOT (ASTM D2872-22) test is mainly used to simulate the short-term thermal oxygen aging phenomenon caused by high temperature and air contact during the actual construction process of asphalt mixing, transportation and paving [24]. RTFOT or the thin film oven test is usually used to simulate the short-term aging process of asphalt [25]. The thin film oven test (TFOT) was the earlier standard for simulating short-term thermal oxidation. It was later superseded by RTFOT, which provides a thinner and more uniform film, ensuring better oxygen contact and more reproducible results. RTFOT is now the internationally recognized standard and was adopted in this study. Firstly, heat the asphalt to flowing state, weigh 35 ± 0.5 g asphalt and pour it into a clean special glass bottle. Insert the glass bottle containing asphalt horizontally into the rotary table in the oven, and keep the open end of the glass bottle facing outward. Set the oven temperature to 163 ± 0.5 °C. After starting the rotary table, the air nozzle continuously blows air into the inside of the glass bottle at a constant rate of 4000 ± 200 mL/min.

#### 2.2.2. Long-Term Thermal Oxidative Aging Test

**Figure 1:**
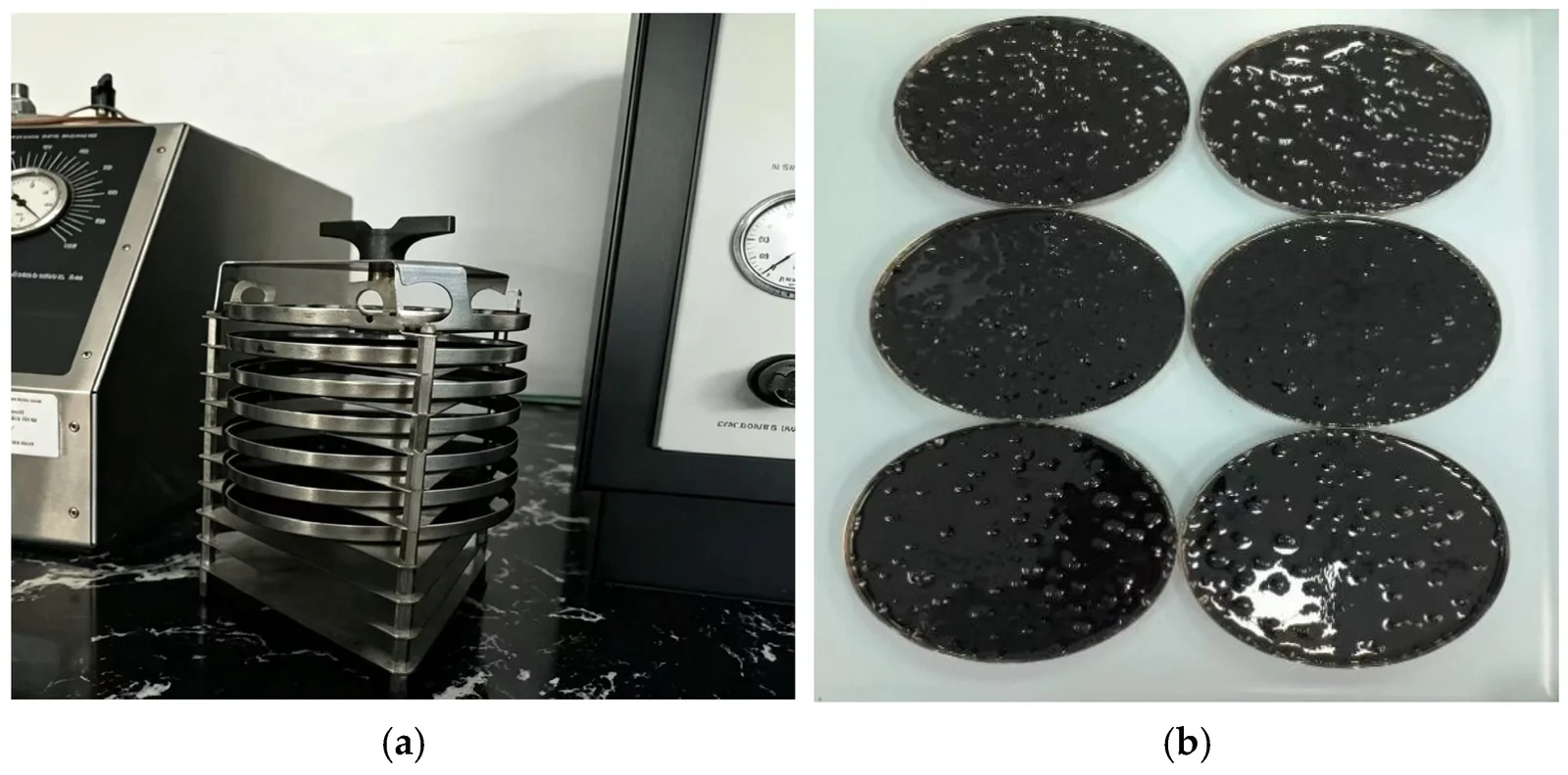
Long-term aging test of asphalt: (**a**) asphalt samples to be aged; (**b**) asphalt samples after long-term aging.

PAV (ASTM D6521-22) is usually used to simulate the long-term aging phenomenon of asphalt after several years of service in actual pavement [26]. The aging of asphalt binders during service is affected by mixture-associated variables such as the volumetric proportions of the mix, permeability of the mix, properties of the aggregates, and possibly other factors. Weigh 50 ± 1 g of asphalt aged in RTFOT and place it in the aging pan. Place the aging tray containing asphalt on the shelf (as shown in Figure 1), and finally put it into the PAV aging box and seal the cover. The test temperature is set at 100 °C, the pressure is set at 2.1 ± 0.1 MPa, and the standard aging time is set at 20 h.

#### 2.2.3. UV Aging Test

Prior to UV aging, all asphalt samples were first subjected to RTFOT aging following the procedure described in Section 2.2.1 to simulate the thermal history during construction. After RTFOT, the aged residue was used for UV exposure. This sequence ensures that the UV aging conditions capture the combined effects of construction-related thermal aging and subsequent field UV radiation. The influence of asphalt in the natural environment is complex. In addition to UV radiation, it is also affected by rain, dust and the alternation of day and night. Reference specification for test method of the asphalt UV aging test: In order to simulate the actual working conditions in the laboratory environment, the equivalent radiation exchange algorithm is used to establish the relationship between indoor test and outdoor solar UV radiation. The calculation formula is shown in Equation (1).

(1) F=Q×T×3600

where *F* is outdoor solar radiation (J/m^2^); *Q* is solar UV radiation intensity (W/m^2^); and *T* is indoor simulated irradiation time (h).

The annual total solar radiation in the Hexi Corridor ranges from approximately 5500 to 6500 MJ/m^2^, with an average value of approximately 5950 MJ/m^2^. The UV component (290–400 nm) typically accounts for about 4–6% of the total solar radiation [27]. Using a conservative estimate of 5%, the annual UV radiation dose is approximately 275–325 MJ/m^2^. Based on the energy equivalence principle (total UV dose = irradiance × time), with a laboratory UV irradiance of 800 W/m^2^ (broadband, 300–400 nm), the time required to accumulate one year’s equivalent UV dose is approximately 104 h. Accordingly, 13 h corresponds to approximately 1.5 months, 26 h to approximately 3.0 months, and 37 h to approximately 4.3 months of equivalent UV exposure. On this basis, 26 h and 37 h were further selected as extended aging nodes to investigate the cumulative degradation effect of UV aging duration on asphalt performance. The appearance of surface oxidation saturation and photo degradation competition mechanism (such as the increase in the AFM bee-like structure area ratio and the non-monotonic change in stiffness modulus at very low temperature) can be observed in the limited test cycle, which is helpful to reveal the nonlinear evolution characteristics of UV aging.

**Figure 2:**
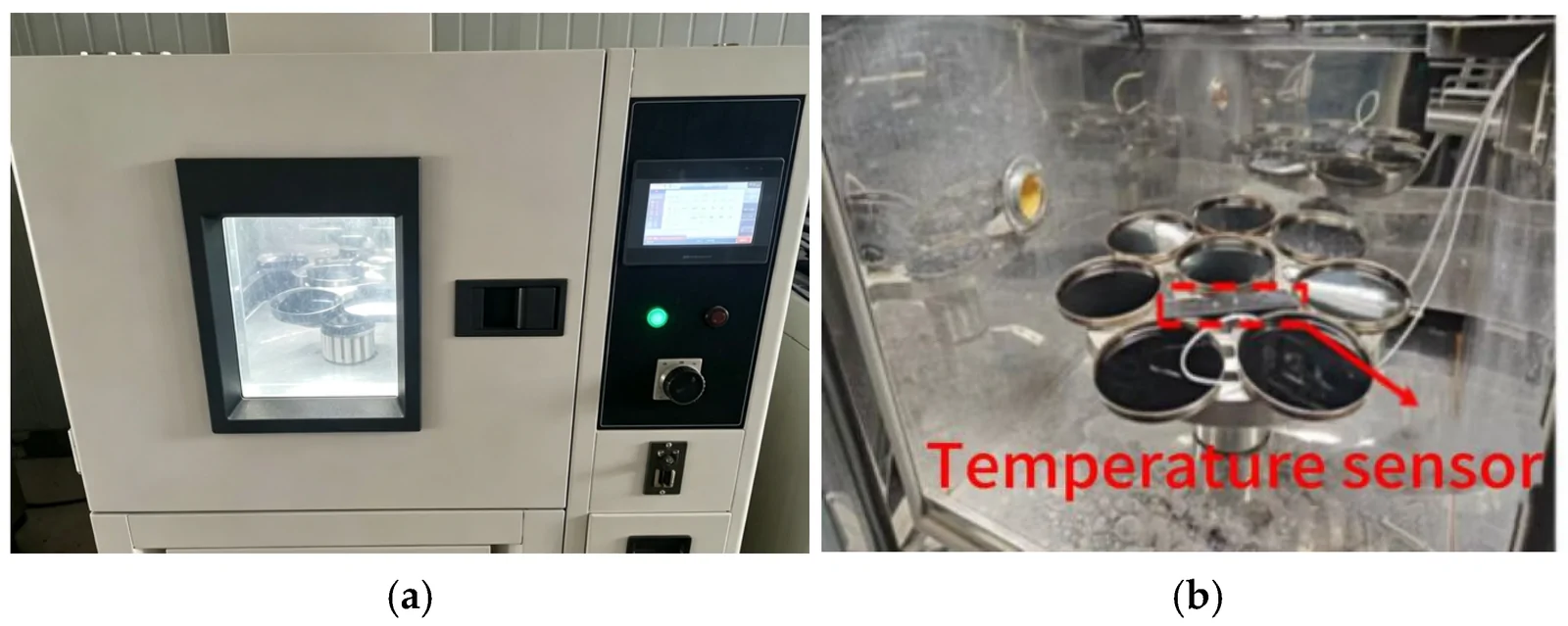
UV aging test of asphalt: (**a**) asphalt UV aging simulation box; (**b**) asphalt samples aging under UV radiation.

Firstly, the sample is subjected to RTFOT to complete the short-term aging. Take 35 ± 0.5 g asphalt after short-term aging and pour it into the asphalt aging tray with a diameter of 14 cm. Heat it in the oven at 135 °C for 20 min to pave it. In order to ensure uniform film thickness, it must be placed strictly horizontally during preparation and aging. Place the sample on the aging turntable in the environmental box of the artificial strong UV light source. The upper layer of the environment box is equipped with UVA-340 fluorescent UV lamps (peak emission at 340 nm, spectral range 295–400 nm) and a ventilator. The broadband UV irradiance (295–400 nm) was set at 40 W/m^2^, measured using a UV-specific radiometer with a 295–400 nm bandpass filter. For reference, the corresponding narrowband irradiance at 340 nm is approximately 0.76 W/(nm·m^2^). It should be noted that the adopted UV irradiance (800 W/m^2^) is approximately one order of magnitude above natural peak levels. The correspondence between laboratory duration and field service time relies on a reciprocity assumption, which may not perfectly hold for asphalt photo-oxidation, as high irradiance could alter reaction kinetics or induce surface saturation effects. Therefore, the equivalent field times reported here should be regarded as estimates for comparative purposes rather than precise predictions of actual field aging. Adjust the asphalt surface temperature to 50 ± 1 °C through the blackboard temperature. The daily irradiation time is set at 13 h. The test equipment and process are shown in Figure 2.

The daily irradiation time was set at 13 h to simulate daytime UV exposure. The 26 h and 37 h aging conditions were accumulated over multiple consecutive days (2 and 3 days, respectively), with dark periods (11 h per day) between each irradiation cycle to simulate diurnal cycles. The asphalt film thickness was approximately 2.2 mm, calculated from the sample mass (35 ± 0.5 g), tray area (diameter 14 cm), and asphalt density (1.02 g/cm^3^). After UV aging, the entire aged asphalt film was scraped from the tray, re-melted at 135–140 °C, and mechanically stirred to ensure homogeneity before molding BBR beams. It should be noted that this reheating step may introduce additional thermal aging, and the surface-aged layer (top tens of micrometers) becomes diluted into the bulk upon re-melting; therefore, the measured properties represent bulk-averaged rather than surface-only responses.

It is worth comparing the above exposure schedule with those adopted in previous UV aging studies. For instance, Yang et al. employed UV aging durations of 3, 6, and 9 days (72, 144, and 216 h) to investigate the long-term rheological response of Sasobit/SBS/nano-TiO_2_-modified binders [28]. In contrast, the relatively shorter durations selected in this study (13, 26, and 37 h) were deliberately chosen to capture early-stage aging kinetics and to establish a mechanistic bridge between UV photo-oxidation and standard thermal aging.

### 2.3. Evaluation of Low-Temperature Rheological Properties

Through the combination of macro-rheological test and micro-detection technology, this study comprehensively evaluated the evolution law of different asphalt under light–temperature coupling.

**Figure 3:**
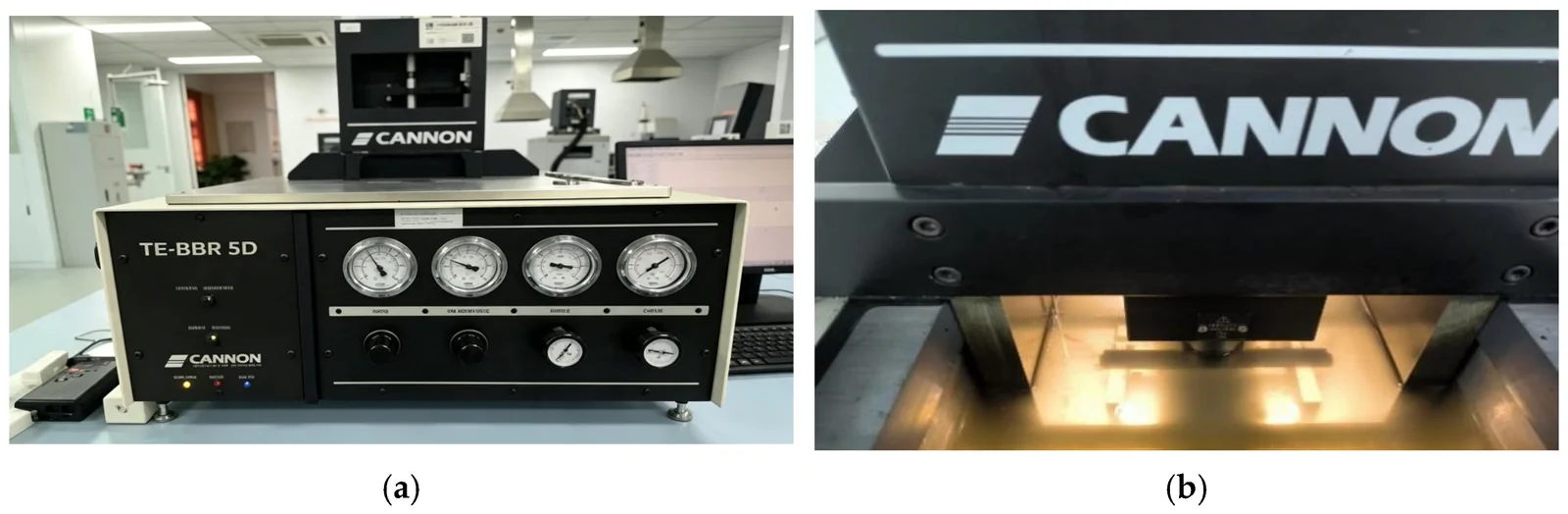
Rheological test of bending beam: (**a**) bending beam rheometer; (**b**) test process.

BBR is used to evaluate the low-temperature crack resistance of asphalt (ASTM D6648-25a) [29]. The test instrument and loading process are shown in Figure 3. Asphalt beams with a size of 127 × 12.7 × 6.35 mm^3^ (ASTM D6648) are prepared and cooled to room temperature and then kept in alcohol medium for 1 h. The test temperature is −12 °C, −18 °C and −24 °C. The low temperature performance is evaluated by measuring the creep stiffness modulus (S) and creep rate (m) at 60 s. The S indicates the ability of the asphalt binder to resist deformation at a low temperature. A higher value means higher rigidity and a higher risk of brittle cracking. The creep rate m-value shows the ability to relax stress with time at a low temperature. A higher value means better stress relaxation and better resistance to cracking. When the stiffness modulus is small and the creep rate is large, it indicates that the asphalt has good low-temperature crack resistance. All BBR tests were performed in triplicate.

### 2.4. Evaluation Methods of Microstructural Properties

(1) AFM

In order to explore the aging mechanism from the microscopic point of view, two physical and chemical analysis methods were used. The AFM is a high-resolution surface morphology characterization tool. AFM uses a nanometer-scale probe to detect the surface topography of a sample; by measuring the forces between the probe and the sample, it determines the height of any point on the surface. AFM imaging was performed using a Bruker Dimension Icon (Billerica, MA, USA) atomic force microscope operating in tapping mode. Commercial silicon probes with a nominal spring constant of approximately 6 N/m and a resonance frequency of approximately 150 kHz were used. All images were acquired over a scan size of 40 μm × 40 μm at a resolution of 512 × 512 pixels. For each sample, at least three images were collected from different locations to ensure representativeness. AFM samples were prepared by hot-droplet deposition. A small amount of asphalt binder was heated to 135–140 °C until fully fluid, and a single droplet was placed on a clean glass slide. The sample was then cooled naturally to room temperature (approximately 23 °C) in air. No solvent was used in sample preparation. All samples were stored in a desiccator for at least 24 h prior to AFM imaging to minimize moisture effects. This preparation protocol was applied consistently to all samples (unaged and aged) to ensure valid comparison.

AFM is used to observe the “honeycomb structure” on the asphalt surface, a characteristic morphology associated with bituminous aggregation. Surface height maps and phase maps are obtained to analyze changes in the number, size, distribution and area fraction of the honeycomb structure during the ageing process. The test method employs the most commonly used tapping mode, in which the tip makes intermittent contact with the sample, thereby minimize damage to the soft asphalt whilst simultaneously acquiring height maps and phase maps. There are many research results on the use of AFM to study the microstructure and related mechanisms of asphalt [30].

(2) FTIR

FTIR is used to monitor the changes in functional groups during aging. Under the irradiation of infrared light, various functional groups in asphalt will produce different vibration absorption, so different chemical bonds will appear in different wave number positions. By analyzing the wave number, peak shape and strength of the spectrum of asphalt samples, we can evaluate the types of and changes in functional groups in asphalt, and then explore the influence of aging on the chemical functional groups of asphalt. FTIR spectrometer (Thermo Scientific™ Nicolet™ iS™5, Waltham, MA, USA) was used to analyze the functional group information of base asphalt under different aging modes, examining asphalt aging mechanisms using FTIR to analyze changes in chemical properties and functional groups. The carbonyl index increases with aging, while the sulfoxide index shows inconsistent growth [31]. FTIR spectra were acquired using a spectrometer equipped with an ATR accessory (diamond/ZnSe crystal). Spectra were recorded over the range of 4000–400 cm^−1^ at a resolution of 4 cm^−1^, with 32 scans co-added per sample and a background scan acquired before each measurement. Automatic baseline correction (rubber-band method) was applied to all spectra. The FTIR measurement was conducted for each sample with the FTIR spectra acquired once per sample. The trends reported are based on the observed spectral changes, which are consistent with established aging mechanisms in the literature.

For quantitative analysis, the carbonyl index (I_C=O_) and sulfoxide index (I_S=O_) were calculated using Equations (2) and (3), where the peak areas were integrated over the following ranges: 1690–1720 cm^−1^ for C=O (carbonyl),1020–1050 cm^−1^ for S=O (sulfoxide), and 1450–1480 cm^−1^ (around 1460 cm^−1^)and 1350–1390 cm^−1^ (around 1376 cm^−1^) for the aliphatic C–H reference peaks. The reference area A_ref_ was taken as the sum of the two aliphatic peak areas. Peak area integration was performed using a straight baseline between the defined integration limits after baseline correction.

FTIR is based on the principle of molecular vibrational spectroscopy. When the infrared light irradiates the sample, the chemical bonds in the molecule (such as C-H, C=O, S=O, etc.) will absorb the infrared light of a specific wavelength, and stretch or bend vibration will occur. Calculation of functional group index: select aliphatic characteristic peaks (about 1460 cm^−1^ and 1376 cm^−1^) as the reference peak area (A_ref_), and calculate the carbonyl index and sulfoxide index [32].

(2) $$I_{C=O}=\frac{A_{C=O}}{A_{ref}}$$

(3) $$I_{S=O}=\frac{A_{S=O}}{A_{ref}}$$

where I_C=O_ is the carbonyl index; I_S=O_ is the sulfoxide index; A_C=O_ is the characteristic peak area of the carbonyl index group; A_S=O_ is the area of the characteristic peak of the sulfoxide index group; A_ref_ is the reference peak area.

**Figure 4:**
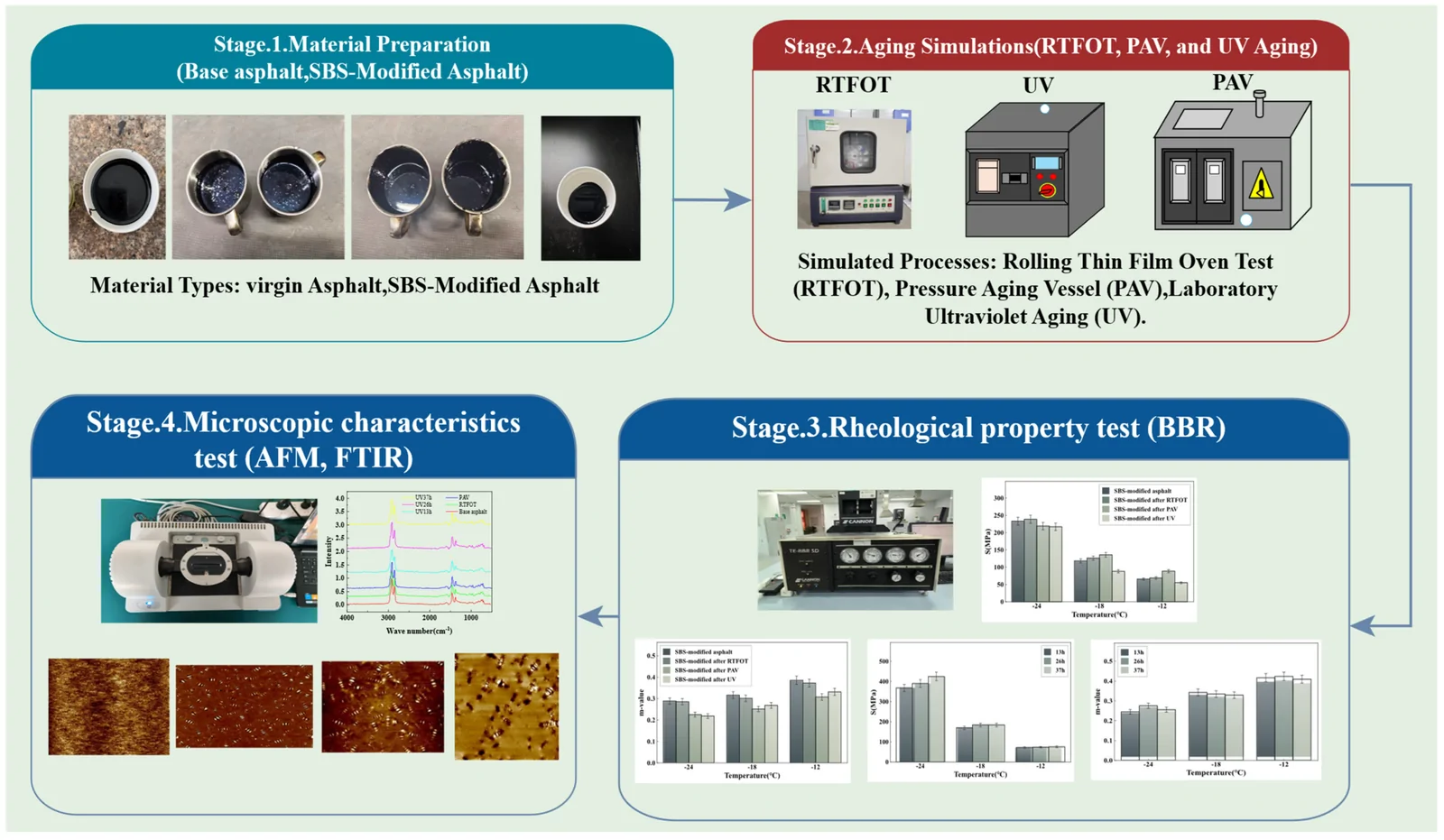
Research methodology flowchart of this study.

Overall, the research methodology flowchart can be summarized as shown in Figure 4, and the evaluation methods and indicators used in this study are summarized in Table 2.

## 3. Results and Discussion

### 3.1. Analysis Low-Temperature Rheological Property

#### 3.1.1. Effect of Different Aging Methods on Low-Temperature Rheological Properties

**Figure 5:**
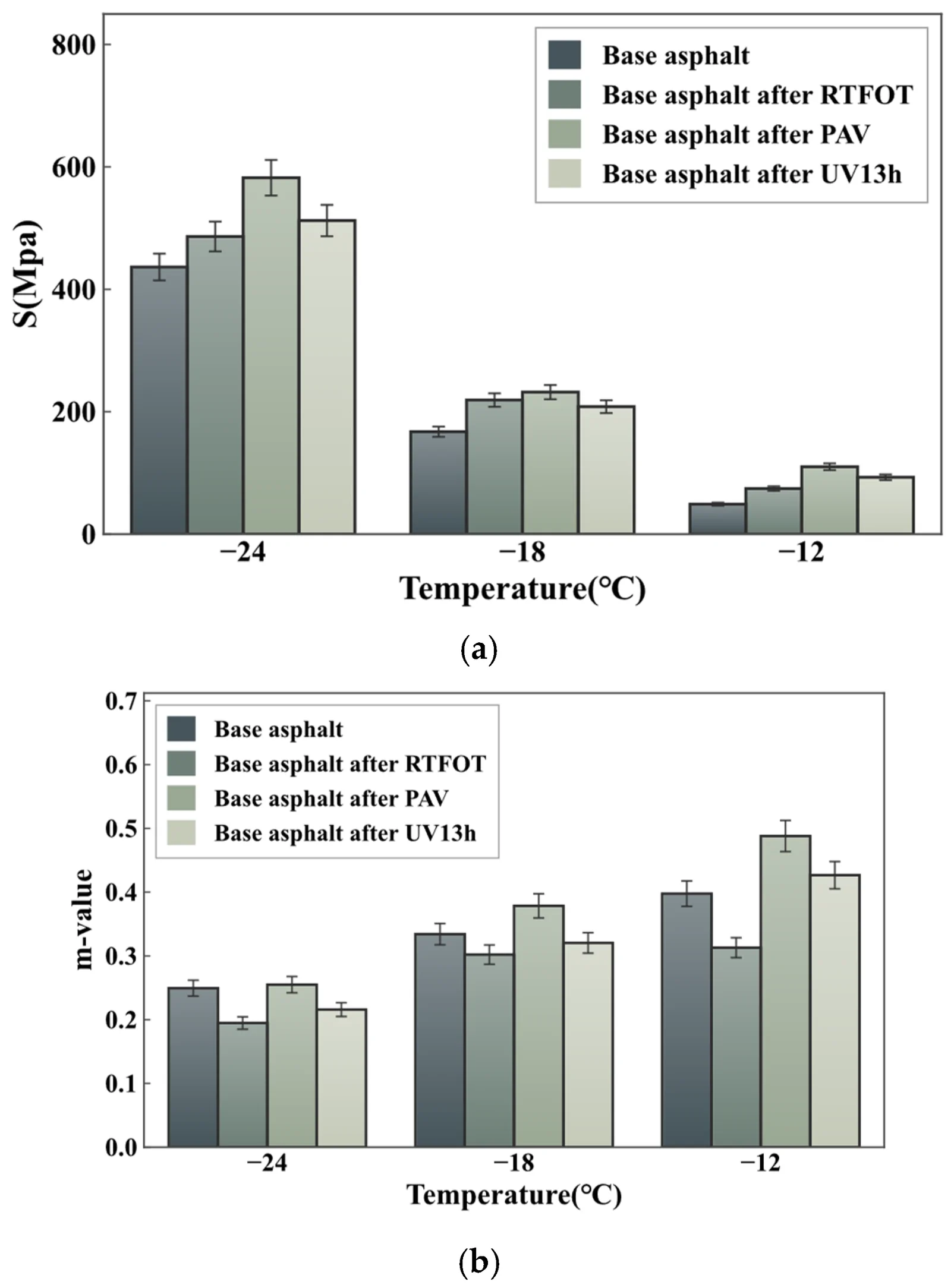
Results of BBR tests on asphalt under different aging conditions: (**a**) S; (**b**) m-value.

Based on the BBR test, the variations in *S* and the m-value for base asphalt under RTFOT aging, PAV aging, and 13 h UV aging are shown in Figure 5, where the testing temperatures are set as −24 °C, −18 °C, and −12 °C, and the error bars represent the standard deviation of three replicate measurements.

As shown in Figure 5, the low-temperature performance of asphalt material is significantly affected by temperature [33]. Within the test range, the S of the four asphalts increased with the decrease in temperature, while the m-value decreased with the decrease in temperature. This is because as the temperature decreases, the asphalt begins to harden, and the elastic component gradually dominates, while the content of the viscous component positively related to the low-temperature performance decreases relatively [34]. Aging will increase the stiffness modulus of the asphalt, reduce the creep rate, and then reduce the ductility of asphalt, which is more prone to brittle fracture at low temperature. The following is a comparison of the effects of different aging methods on low-temperature performance [35].

As shown in Figure 5, the stiffness modulus S for four different aging conditions increases monotonically with the decrease in temperature, and the creep rate m decreases monotonically with the decrease in temperature. For S values, PAV is greater than RTFOT, close to UV for 13 h, and greater than the original sample. The S value of the PAV sample jumps to about 580 MPa at −24 °C, and the m-value appears to undergo obvious “collapse” at −12 °C to −18 °C; the RTFOT and UV-13 h curves exhibit comparable trends across the whole temperature range, suggesting that UV exposure equivalent to approximately one month of field service does not induce additional deterioration beyond that of RTFOT aging. However, this observation is based on trend comparison rather than formal statistical testing, and the two aging mechanisms (bulk thermal oxidation vs. surface photo-oxidation) remain fundamentally distinct. The distinct response of PAV-aged asphalt can be tentatively attributed to changes in its colloidal balance. Under the high-pressure and high-temperature conditions of PAV aging, oxygen molecules diffuse into the bulk of the asphalt film, promoting oxidative polycondensation. It is hypothesized that this process may induce a conversion of resins to asphaltenes, accompanied by the formation of polar functional groups (e.g., carbonyl and sulfoxide) that could further associate through hydrogen-bond networks. Such structural changes would restrict molecular segmental mobility and stress relaxation, resulting in the highest S and lowest m-values observed for the PAV condition. To sum up, Figure 5 reveals the difference in the depth of damage to the asphalt colloidal structure by different aging methods from the perspective of low-temperature rheological response; namely PAV causes bulk oxidation and structural networking, while UV-13 h only causes photooxidation on the surface, and its aging depth is basically equivalent to RTFOT, which provides a direct test basis for the “non-significant effect” of the monthly magnitude of UV radiation service on asphalt low-temperature performance in the high-intensity UV region. The stiffness modulus of base asphalt after PAV is considerably higher than that after short-term aging and UV aging, and the creep rate is the smallest. The performance of stiffness modulus and creep rate of base asphalt after 13 h UV aging is very close to that after short-term aging. This shows that the UV aging amount of pavement in service for one month will not significantly increase the aging degree of asphalt and has little effect on the low-temperature performance of asphalt [36].

**Figure 6:**
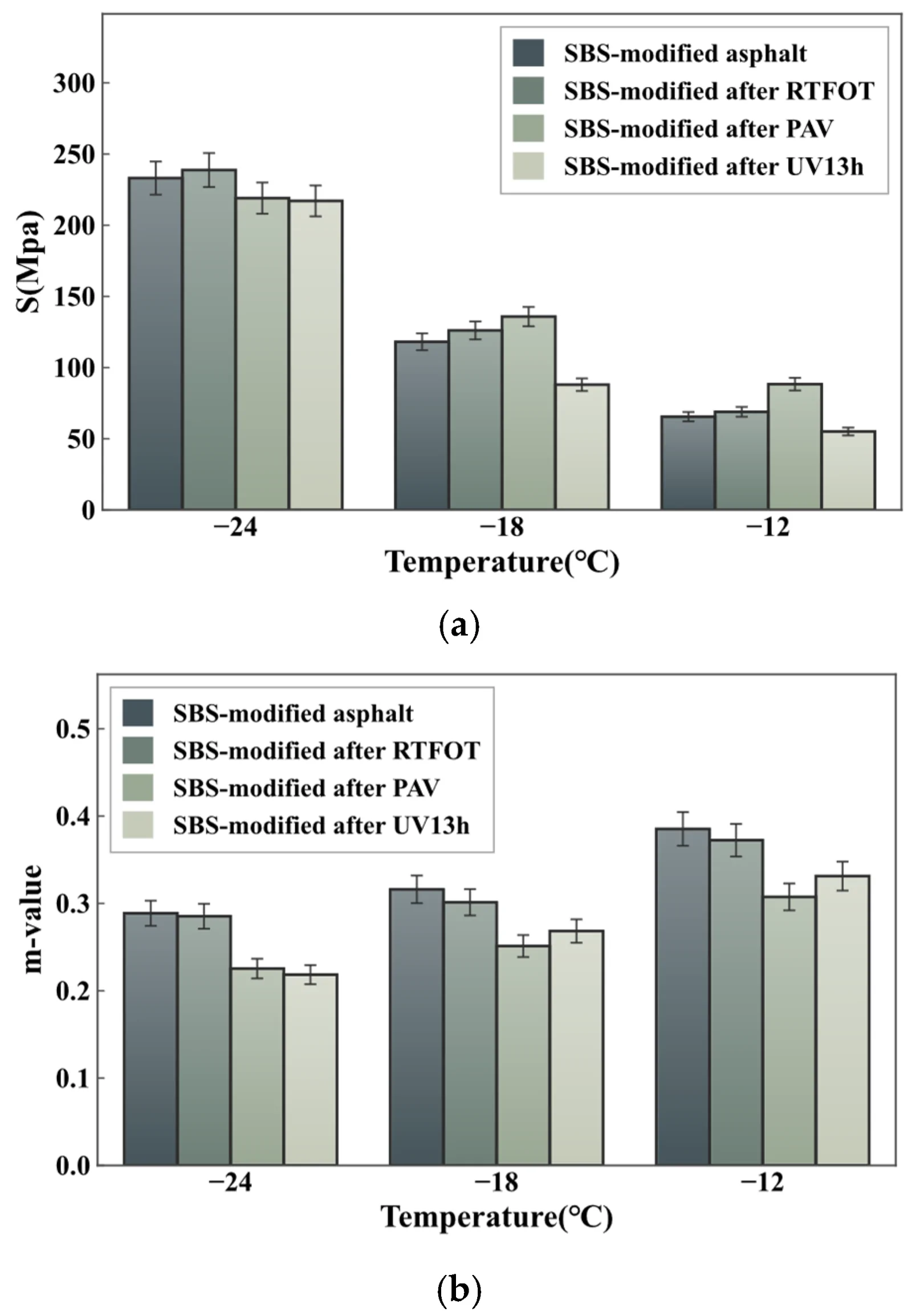
Results of the BBR test for SBS-modified asphalt subjected to different aging methods: (**a**) S; (**b**) m-value.

Moreover, the variations in S and m-value for SBS-modified asphalt under RTFOT aging, PAV aging, and UV aging are compared as shown in Figure 6.

As shown in Figure 6, the low-temperature mechanical response of SBS-modified asphalt shows significant temperature dependence and aging mode difference. Under any specified aging state, with the test temperature dropping from −12 °C to −24 °C, the S value of each sample shows a steep monotonic upward trend, while the m-value has an obvious linear downward trend, which is similar to that of the base asphalt. This indicates that the very-low-temperature environment will directly harden the asphalt and weaken its flexibility.

Within different temperature ranges, short-term aging has little effect on the S value and m-value of SBS-modified asphalt. PAV aging increases the S value and significantly reduces the m-value. At −18 °C and −12 °C, UV aging leads to a progressive increase in S and a decrease in m with extended UV duration, indicating continuous stiffening and loss of stress relaxation capacity. The effect of 13 h UV aging on the S value even exceeds that of PAV aging at these temperatures. Compared with base asphalt, the S value of SBS-modified asphalt shows poorer regularity under PAV aging, while decreasing under UV aging. The m-value decreases more severely. This may be mainly attributed to the SBS modifier. The hard segment of polystyrene in SBS forms a physical crosslinking point in asphalt, and the soft segment of poly butadiene adsorbs aromatic components to form a continuous elastic network. This multi-scale collaborative structure of “hard segment and soft segment rubber particles” can not only maintain sufficient chain relaxation capacity at low temperature, but also delay the diffusion channel of oxygen molecules through the network structure in the bulk phase, thus inhibiting the degree of oxidative polycondensation in the PAV stage, significantly limiting the formation of polar functional groups such as carbonyl and sulfoxide groups.

#### 3.1.2. Effect of Different UV Aging Durations on Low-Temperature Rheological Properties

**Figure 7:**
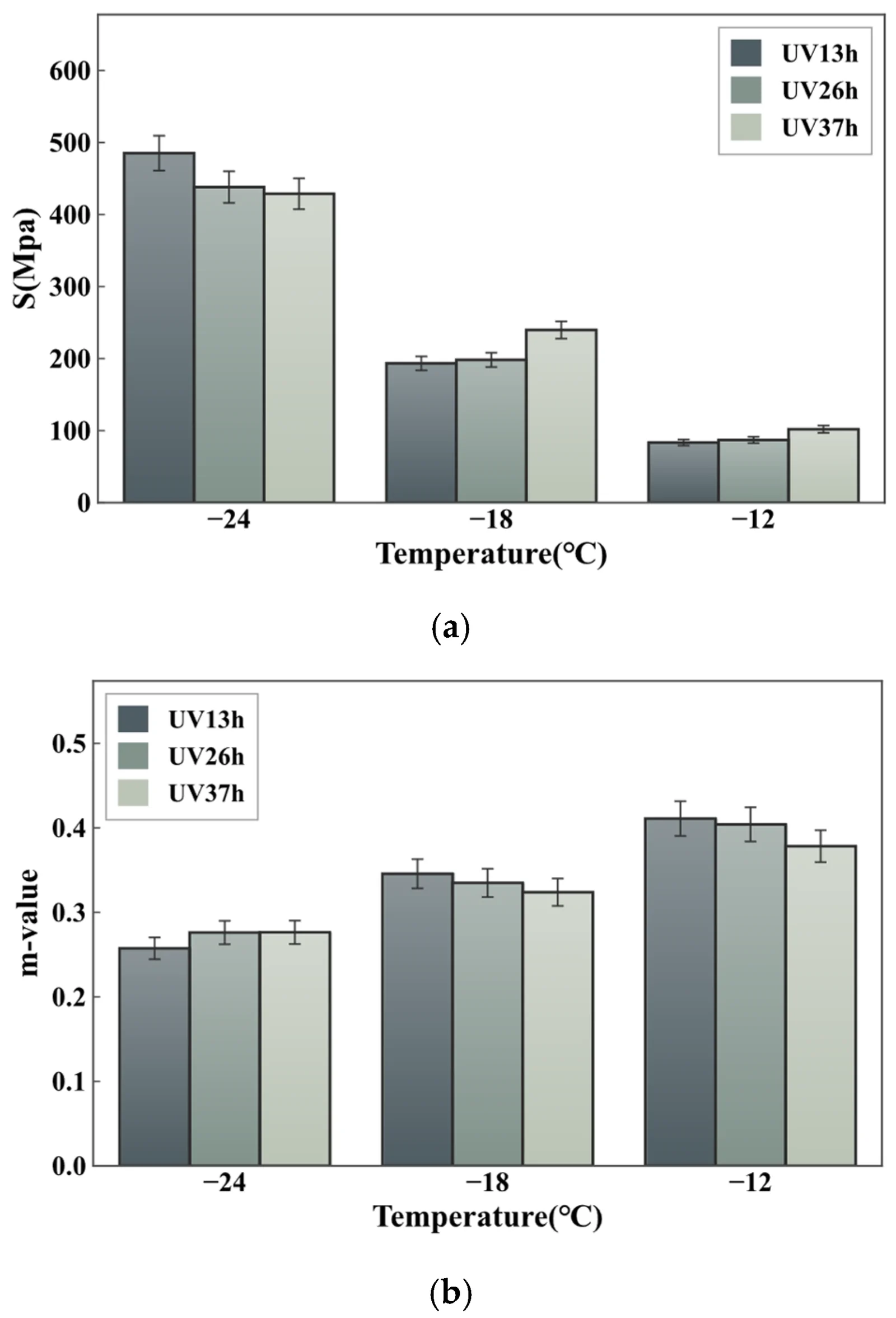
BBR test results for base asphalt subjected to UV aging for 13 h, 26 h, and 37 h: (**a**) S; (**b**) m-value.

To further analyze the effect of UV aging duration, the changes in low-temperature rheological properties of base asphalt under UV aging for 13 h, 26 h, and 37 h were measured, as shown in Figure 7.

As shown in Figure 7, the S value continues to rise and the m value continues to decline at −12 °C and −18 °C with increasing UV aging time from 13 h to 37 h, indicating that low-temperature crack resistance deteriorates progressively and exhibits clear time dependence. At the extremely low temperature of −24 °C, however, an apparent non-monotonic trend is observed: the S value under 13 h UV is the highest, followed by 26 h, and the lowest under 37 h, while the m value slightly increases from 0.26 at 13 h to 0.28 at 37 h. This apparent recovery, though possibly indicative of UV-induced mechanisms, should be interpreted with caution, as the error bars at this temperature show some overlap (triplicate measurements). The trend may suggest a feature associated with UV aging, but further verification is needed. The UV-13 h dataset in Figure 5 and Figure 7 originates from the same experimental measurements (mean ± SD, n = 3). The slight visual differences in bar heights arise from different y-axis scaling between the two figures.

**Figure 8:**
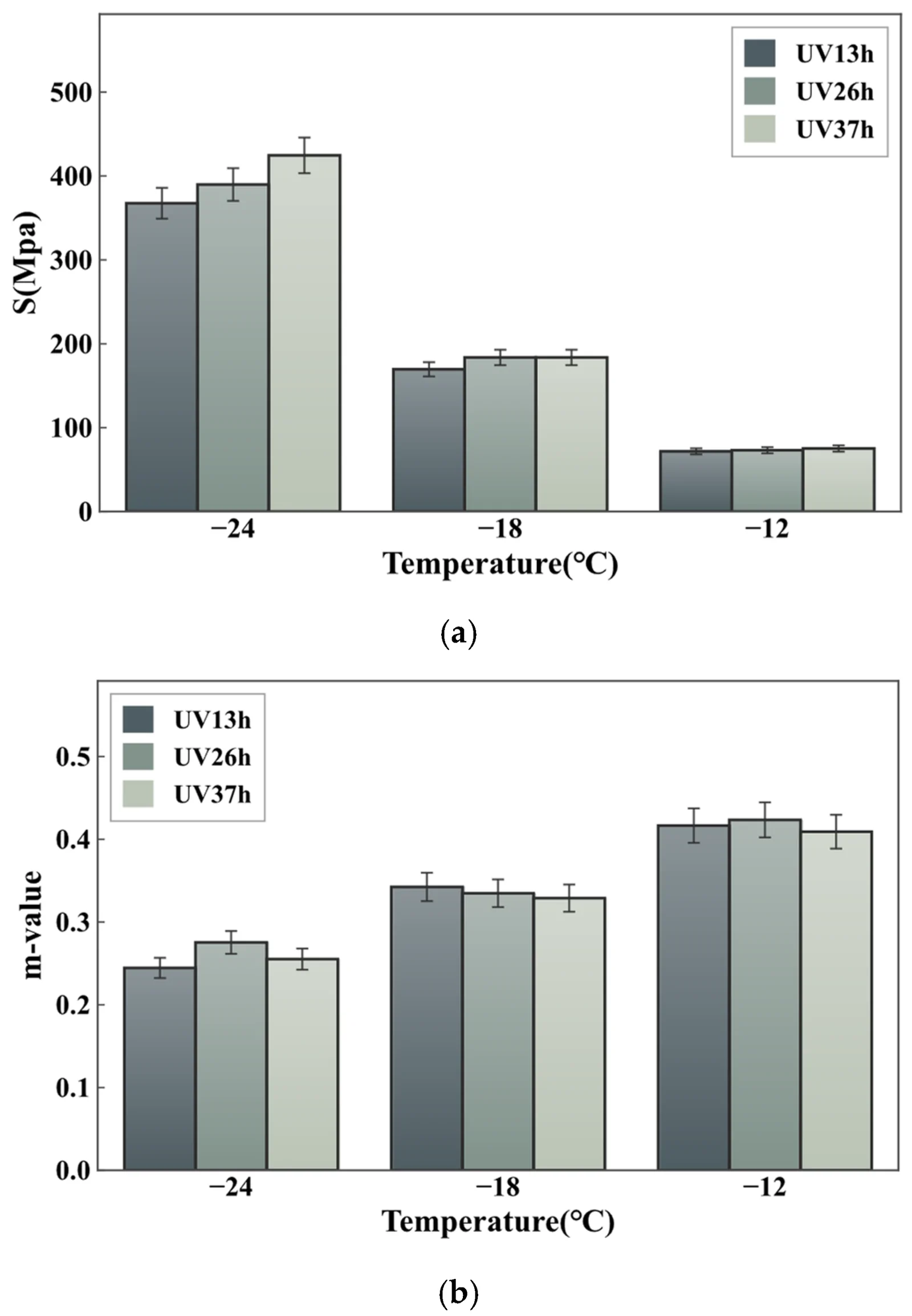
Results of the BBR test for SBS-modified asphalt subjected to UV aging for 13 h, 26 h, and 37 h: (**a**) S; (**b**) m-value.

Moreover, the low-temperature rheological properties for SBS-modified asphalt under UV aging for 13 h, 26 h, and 37 h were also compared, as shown in Figure 8, where error bars represent the standard deviation of three replicate measurements.

As shown in Figure 8, the low-temperature performance of asphalt shows a further decline trend with the extension of UV aging time. The S value of SBS-modified asphalt is the largest after UV aging for 37 h, and the creep stiffness modulus of SBS-modified asphalt is the smallest after UV aging for 13 h. Although there is little difference between the creep stiffness modulus of SBS-modified asphalt after aging for 26 h and 37 h at −18 °C, in general, the creep stiffness modulus of SBS-modified asphalt increases with the increase in aging time, that is the low-temperature performance of SBS-modified asphalt decreases and worsens with the increase in aging time. At −12 °C and −18 °C, the m-value decreases monotonically with UV duration, confirming progressive loss of stress relaxation capacity. At −24 °C, however, the m-value follows a non-monotonic pattern: it increases from 0.245 at 13 h to 0.278 at 26 h, then decreases to 0.257 at 37 h. This indicates that the aging response at extremely low temperatures is less straightforward and may involve competing mechanisms. The statement that “the creep rate was lowest after 37 h” is not supported by the data at −24 °C and has been removed.

### 3.2. Analysis of Micro Characteristic Evolution Morphology

#### 3.2.1. Microscopic Morphology Evolution Based on AFM

(1) Effect of different aging methods

**Figure 9:**
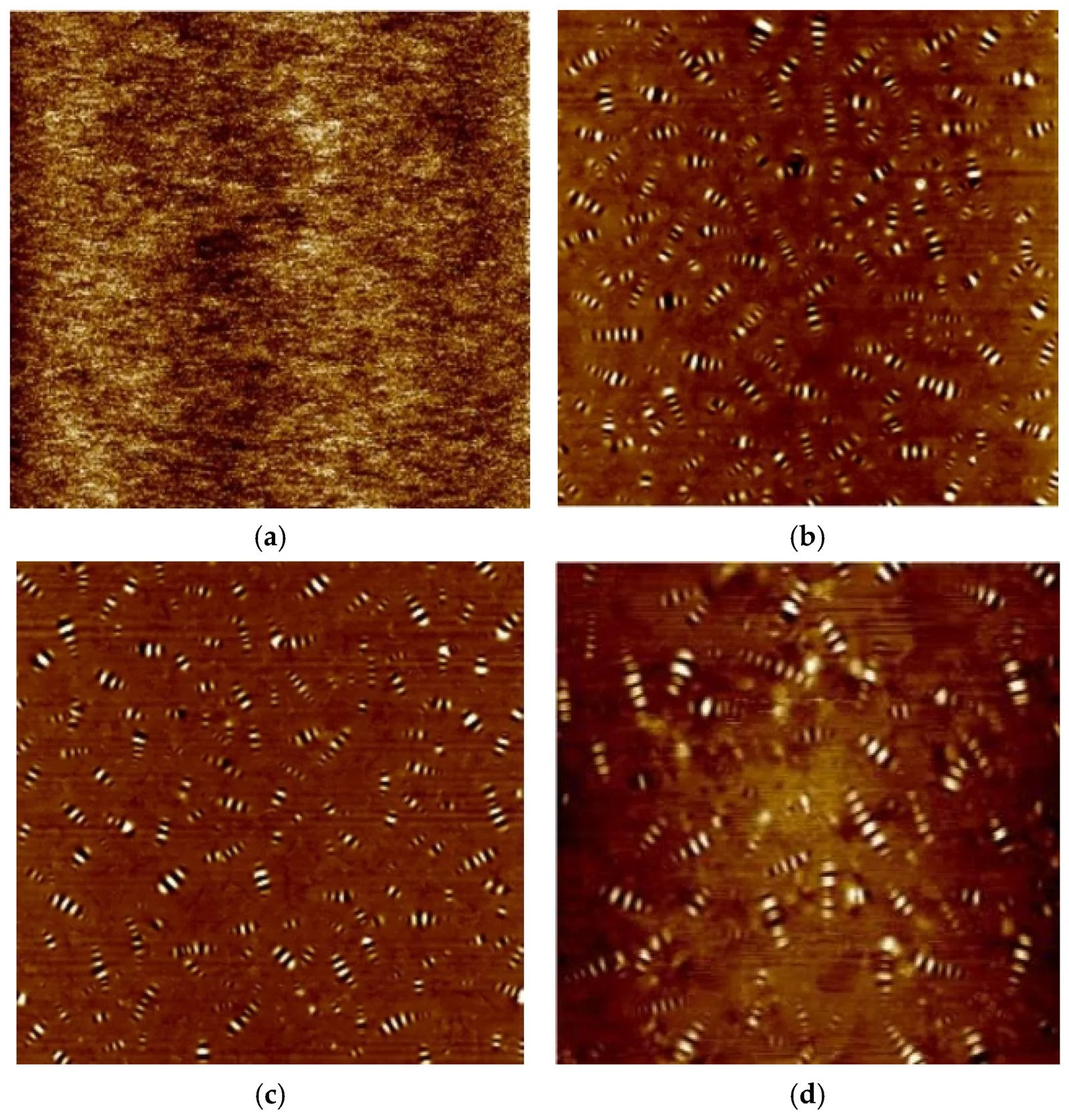
Two-dimensional AFM morphology of the base asphalt: (**a**) base asphalt; (**b**) PAV; (**c**) RTFOT; (**d**) 13 h UV, where the scan size is 40 μm × 40 μm.

Based on the AFM test, the variations in the two-dimensional morphology of the base asphalt with different aging methods are shown in Figure 9.

As shown in Figure 9, there are significant differences in the AFM morphology of base asphalt under different aging methods. When the asphalt is not aged, the surface of the base asphalt is flat without obvious “bee-like structure” [37]. After RTFOT and PAV aging, the surface distribution has a clear “bee-like structure”, and with the deepening of aging, the bee-like structure tends to aggregate and the size increases.

**Figure 10:**
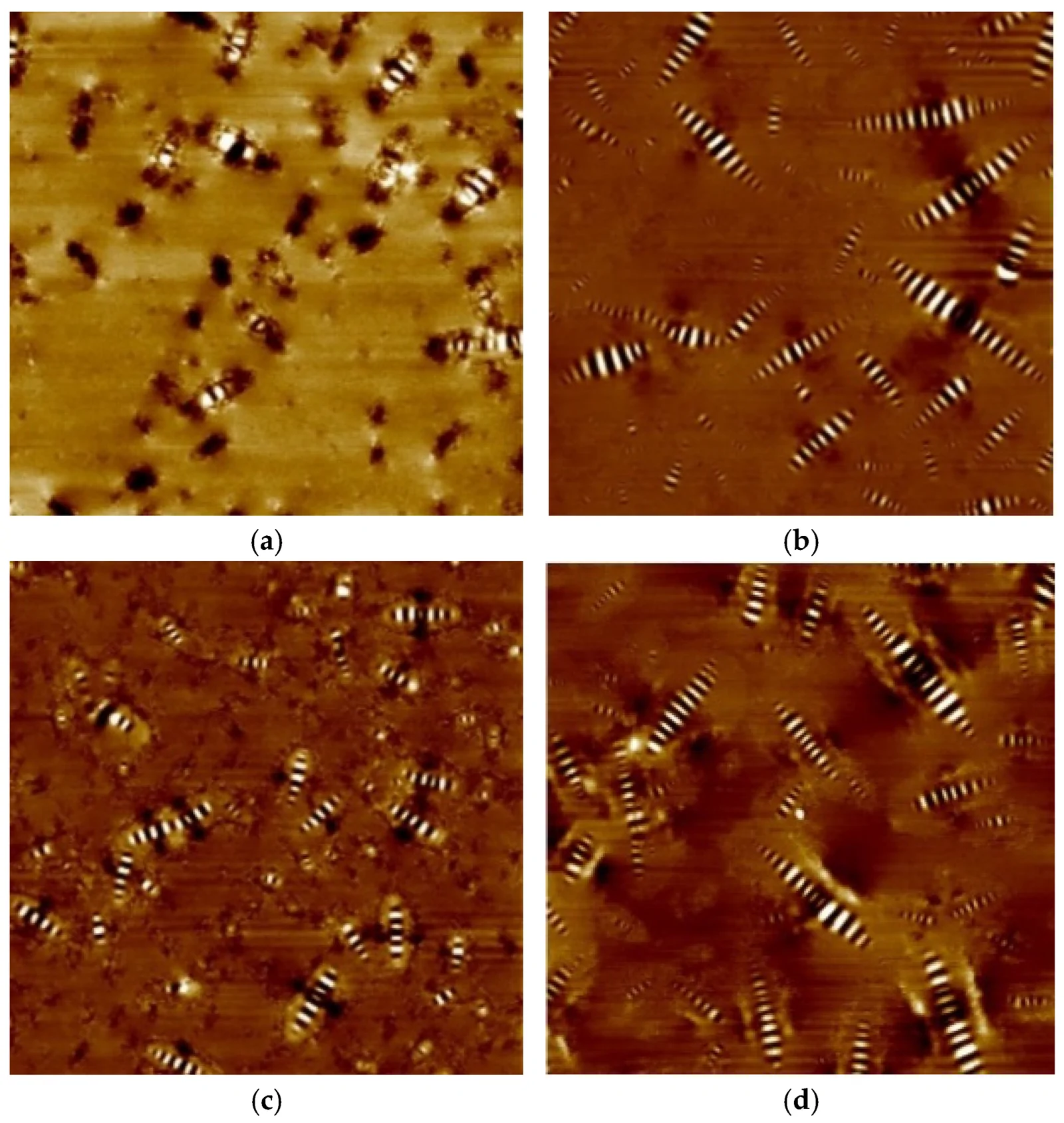
Two-dimensional AFM morphology of SBS-modified asphalt: (**a**) SBS-modified asphalt; (**b**) PAV; (**c**) RTFOT; (**d**) UV, where the scan size is 40 μm × 40 μm.

Comparatively, the variations in the two-dimensional morphology of the SBS-modified asphalt with different aging methods are shown in Figure 10.

As shown in Figure 10, SBS-modified asphalt exhibits more dispersed bee-like structures in the unaged state compared to base asphalt, and its morphological features are less pronounced. After aging, the number of bee-like structures increases, but remains far lower than that of base asphalt under the same aging conditions. Previous studies have shown that SBS-modified asphalt exhibits minimal changes in rheological properties following short-term aging, and that the trends in bee-structure evolution vary with aging conditions [38]. The reason may be that SBS-modifier particles absorb some light components, and the macromolecular structures in SBS particles crosslink with each other, forming solvated layers that overlap and thereby inhibit the growth of bee-like structures.

(2) Effect of UV aging durations

**Figure 11:**
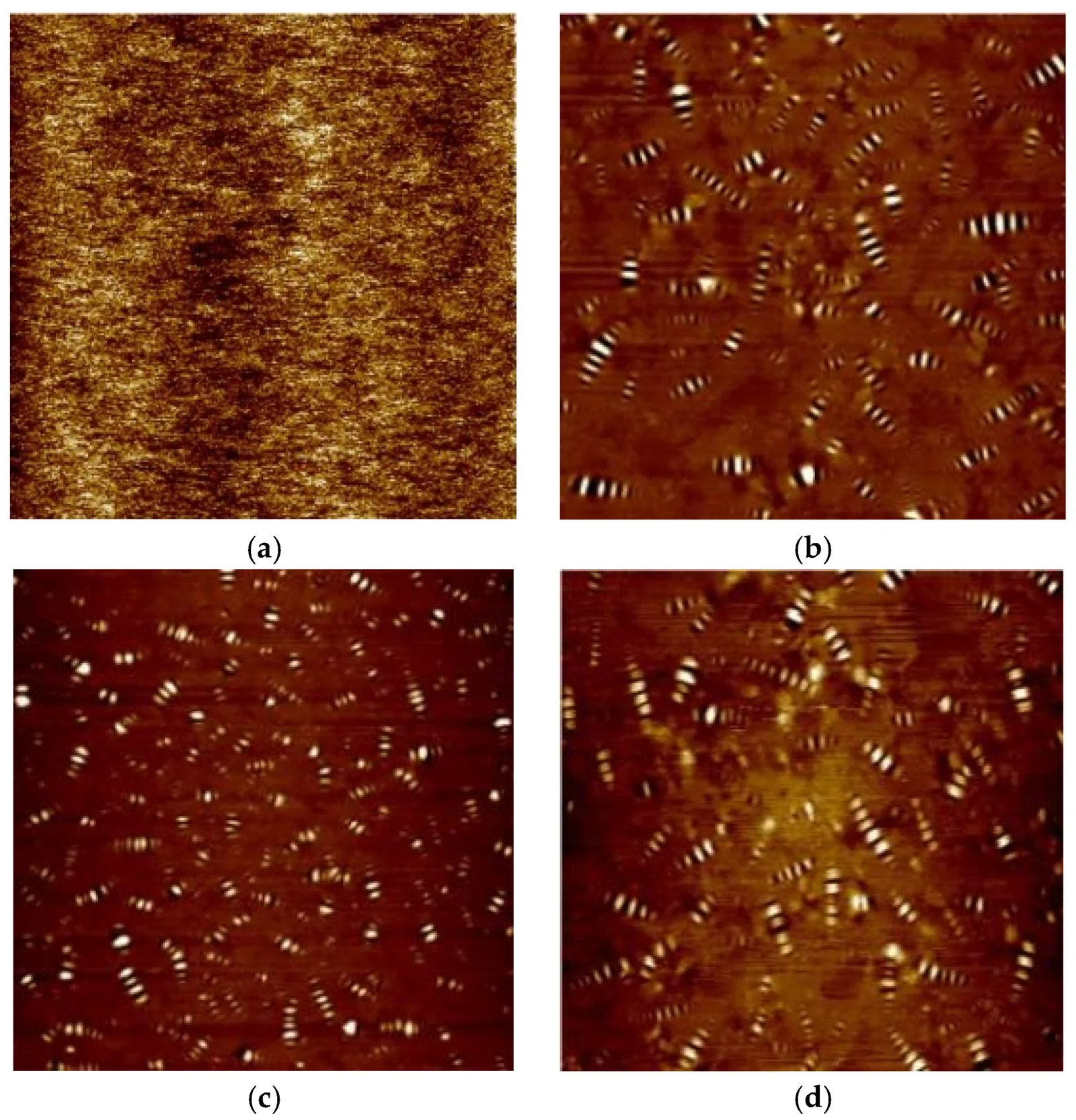
Two-dimensional AFM morphology of base asphalt at different aging duration: (**a**) 0 h UV; (**b**) 13 h UV; (**c**) 26 h UV; (**d**) 37 h UV, where the scan size is 40 μm × 40 μm.

As seen from Figure 11, the number of bee-structures and bee area ratio in asphalt increased after UV aging. With the increase in UV aging time, qualitatively, the number and area fraction of bee-like structures tended to increase initially and then decrease with prolonged UV exposure. However, these observations are based on visual inspection of representative AFM images rather than formal quantitative statistical analysis; therefore, they should be interpreted as qualitative trends rather than definitive conclusions. With increasing UV aging time, resins are continuously converted into asphaltenes, leading to an initial increase in the number of bee-like structures and a sharper, more refined morphology. However, as asphaltene content increases beyond a certain threshold, further UV exposure may induce the decomposition or restructuring of asphaltene molecules, accompanied by the volatilization of lighter molecular components [39], resulting in a subsequent decrease in both the number and area fraction of bee-like structures.

**Figure 12:**
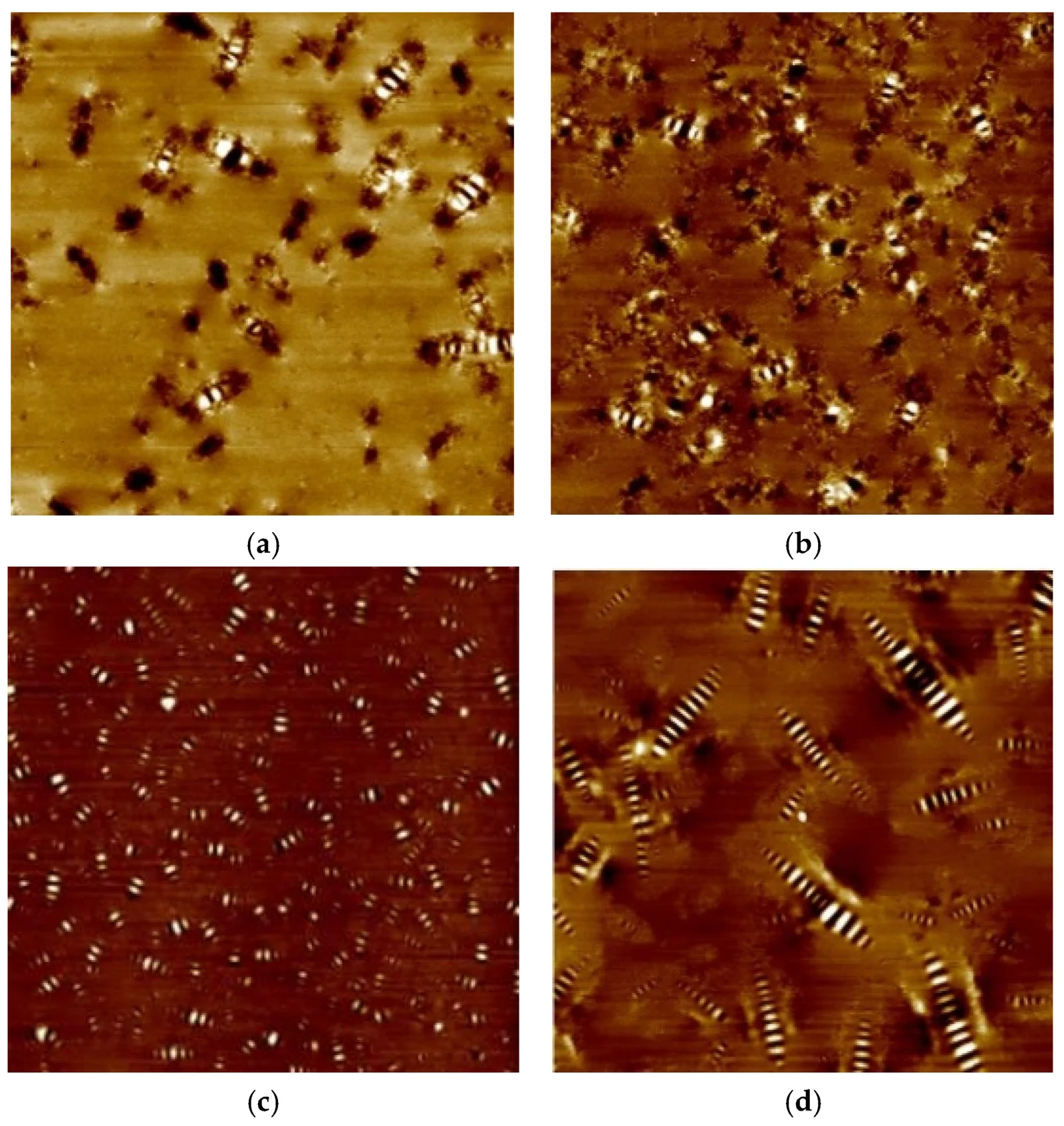
Two-dimensional AFM morphology of SBS-modified asphalt at different aging duration: (**a**) 0 h UV; (**b**) 13 h UV; (**c**) 26 h UV; (**d**) 37 h UV, where the scan size is 40 μm × 40 μm.

It can be seen from Figure 12 that the number of bee-like structures and bee area ratio of SBS-modified asphalt increase first and then decrease with the increase in UV aging time, which is consistent with the change law of the above base asphalt. During the UV aging process of SBS-modified asphalt, there may be not only condensation of base asphalt and the migration of components, but also potential degradation of the SBS modifier; however, this is a tentative interpretation in the absence of SBS-specific FTIR indices. From the change rule of the number of bee-structures, SBS-modified asphalt is similar to base asphalt in the early stage of aging, which is mainly based on the migration of the components of base asphalt, which shows that the number of bee-structures increases, and the bee-structures show a trend of becoming thinner and sharper.

Based on observation of base asphalt (Figure 11) and SBS-modified asphalt (Figure 12) under different UV aging times (0 h, 13 h, 26 h, and 37 h), it is found that the number and area fraction of bee-like structures of base asphalt tended to increase initially and then decrease with increasing UV aging duration. This observation is based on visual inspection of representative AFM images and should be interpreted as a qualitative trend rather than a definitive conclusion.

It should be noted that the SBS-specific polybutadiene (≈966 cm^−1^) and polystyrene (≈699 cm^−1^) bands were not quantified in this study; therefore, interpretations involving SBS polymer degradation remain speculative.

#### 3.2.2. Evolution of Chemical Functional Groups Based on FTIR

**Figure 13:**
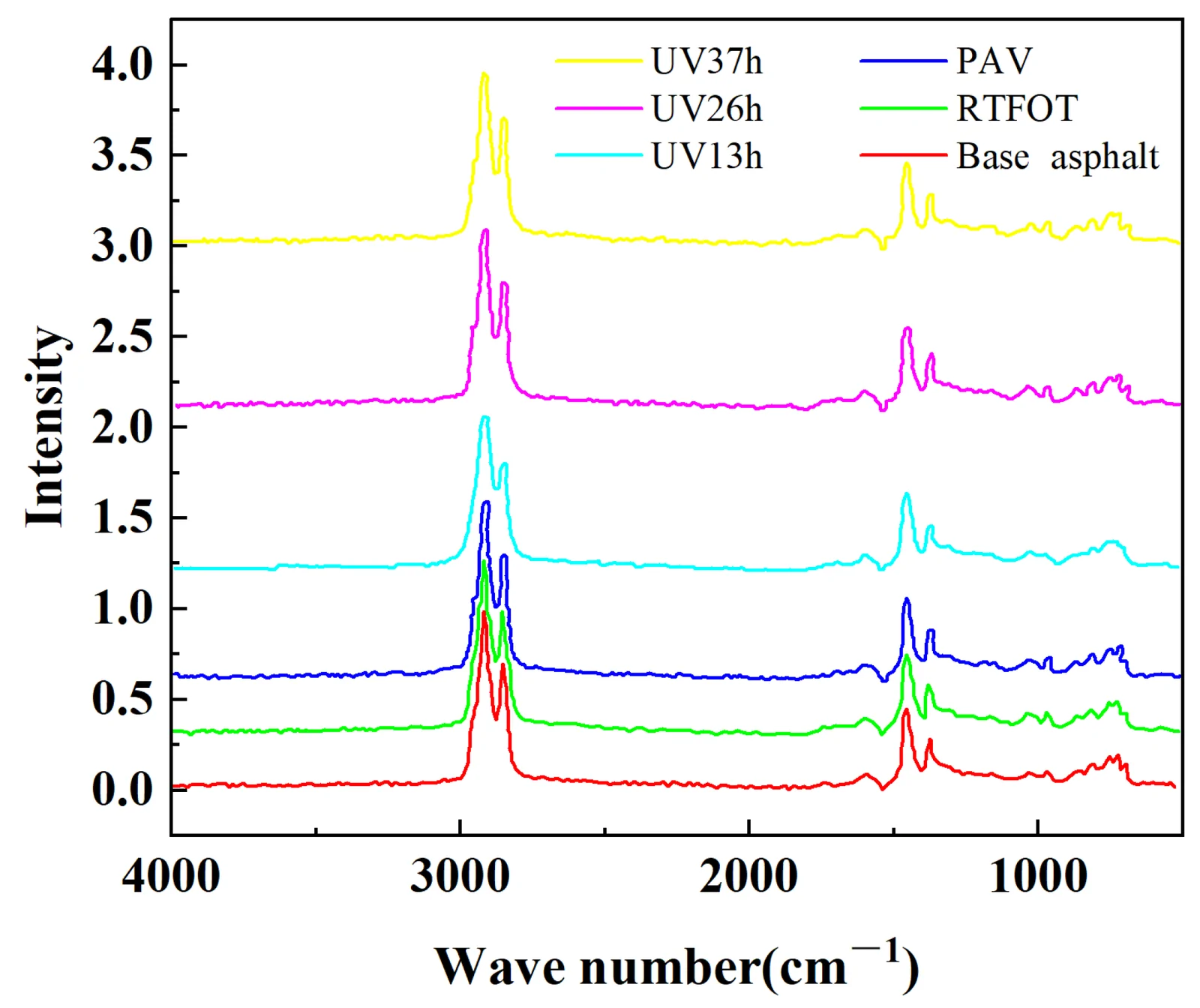
Infrared spectra of base asphalt under different aging conditions.

**Figure 14:**
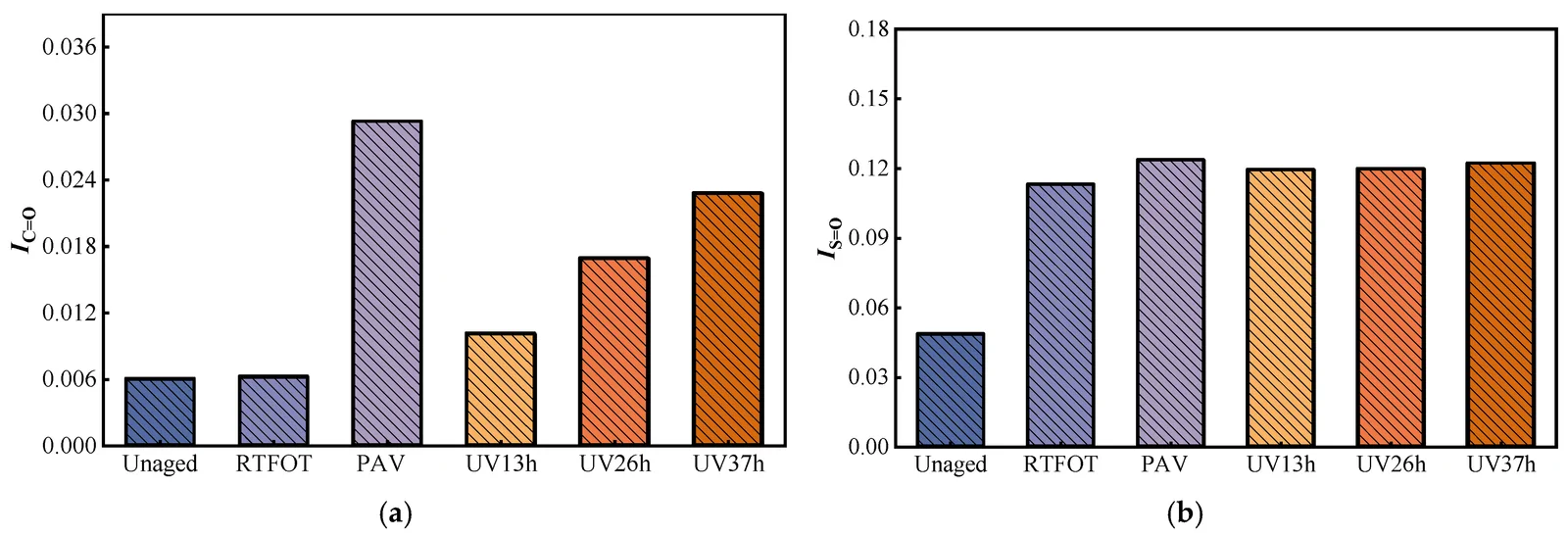
Functional group indexes of base asphalt under different aging conditions: (**a**) I_C=O_ of base asphalt under different aging conditions; (**b**) I_S=O_ of base asphalt under different aging conditions.

FTIR can be used to obtain the corresponding wave numbers of infrared spectra of different aging methods for base asphalt, as shown in Figure 13. Moreover, according to the FTIR test results, the carbonyl index (I_C=O_) and sulfoxide index (I_S=O_) of base asphalt under different aging regimes were calculated using Equations (2) and (3) as shown in Figure 14. The chemical nature of morphological changes in the asphalt surface structure during the aging process was clarified by functional group analysis. The aging time was 13 h, 26 h and 37 h, respectively.

As shown in Figure 13, comparing the infrared spectra of base asphalt under different early aging modes, it can be seen that the position and shape of the characteristic absorption peaks of the unaged base asphalt and those after different aging modes change very little, with only differences in the intensity of some characteristic absorption peaks. The characteristic absorption peak intensities near 2920 cm^−1^ and 2850 cm^−1^ are relatively high, but there is little change in intensity before and after various aging modes. Unaged asphalt did not show obvious I_C=O_ characteristic peaks at 1700 cm^−1^, and the intensity of the I_S=O_ characteristic peak near 1030 cm^−1^ was also low [40]. However, after different modes of aging, obvious I_C=O_ and I_S=O_ characteristic peaks appeared, indicating that asphalt underwent oxidation reactions during the aging process, resulting in the formation of I_C=O_ and I_S=O_ functional groups, which have an effect on the macroscopic properties of asphalt. Overall, base asphalt did not show any new characteristic absorption peaks before and after aging, indicating that no new functional groups were generated during the aging process.

As shown in Figure 14, the aging effect of asphalt can be evaluated by monitoring the changes in I_C=O_ and I_S=O_ content. Due to the diffusion of oxygen molecules in asphalt, oxidative polycondensation will occur in asphalt under a thermal environment, resulting in an increase in I_C=O_ content and a decrease in carbon double bond (C=C) and I_S=O_ (S=O) content. It can be seen from Figure 14 that I_C=O_ and I_S=O_ gradually increase with the deepening of aging degree. After RTFOT aging, the I_C=O_ and I_S=O_ of base asphalt increased by approximately 3.2% and 132.8%, respectively, relative to the unaged sample. After PAV aging, I_C=O_ increased by approximately 388.4%, while I_S=O_ increased by approximately 361.6%, reflecting the more severe oxidation induced by long-term thermal aging. The I_C=O_ and I_S=O_ of base asphalt increased after aging in different aging modes. In terms of I_C=O_, short-term aging causes limited improvement in I_C=O_, while long-term aging results in significant improvement in the I_C=O_ of base asphalt.

**Figure 15:**
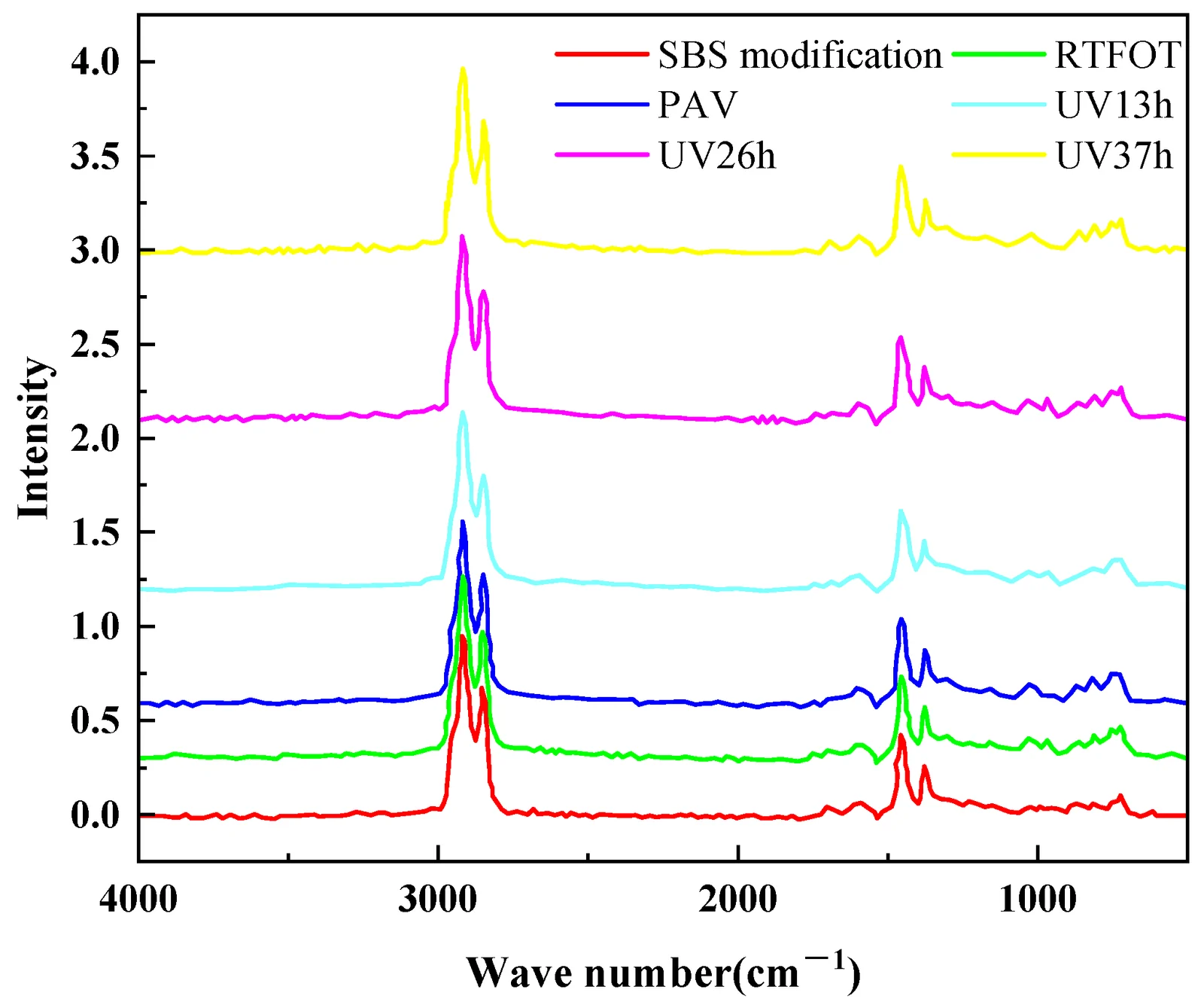
Infrared spectra of SBS-modified asphalt under different aging conditions.

**Figure 16:**
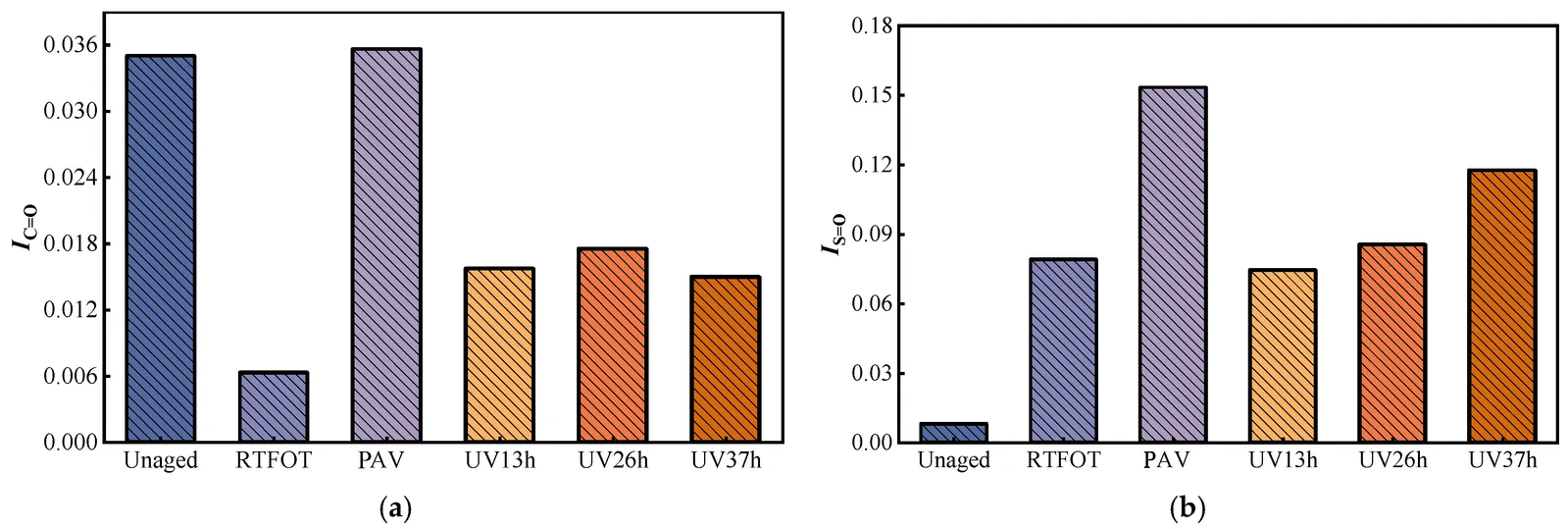
Functional group indexes of SBS-modified asphalt under different aging conditions: (**a**) I_C=O_; (**b**) I_S=O_.

Moreover, the FTIR infrared spectra results for SBS-modified asphalt are shown in Figure 15. The corresponding I_C=O_ and sulfoxide indexes were also calculated as shown in Figure 16.

As shown in Figure 15, the infrared spectrum of SBS-modified asphalt is generally similar to that of base asphalt, with differences in some characteristic absorption peaks. Compared to base asphalt, the characteristic absorption peak of unaged SBS-modified asphalt disappears at 1740 cm^−1^, but the intensity of the I_C=O_ characteristic peak increases at 1700 cm^−1^. After different aging modes, SBS-modified asphalt showed obvious characteristic peaks of I_S=O_ groups compared to unaged SBS-modified asphalt, indicating that SBS-modified asphalt produced a large number of I_S=O_ functional groups due to oxidation reactions during the aging process.

As shown in Figure 16, the I_S=O_ of SBS-modified asphalt increases with the deepening of aging, while the I_C=O_ first increases and then decreases. Compared with base asphalt, the functional group index of SBS-modified asphalt is generally lower, indicating that its anti-aging performance is better. The I_C=O_ of SBS-modified asphalt exhibits a non-monotonic pattern under UV aging, showing an initial increase followed by a decrease. However, the absolute I_C=O_ values for the SBS-modified asphalt should be interpreted with caution, as the baseline correction for SBS-containing binders is complicated by the contribution of the polymer itself. Therefore, the discussion of I_C=O_ for SBS-modified asphalt is limited to trend-based observations rather than quantitative comparisons. The sulfoxide index (I_S=O_), which is less affected by baseline artifacts, shows a clear monotonic increase with aging severity and is used as the primary chemical indicator for SBS-modified asphalt in this study. Under UV aging conditions, the SBS-modified asphalt exhibits a lower total functional group index (I_C=O_ and I_S=O_) than base asphalt, indicating better overall anti-oxidative capacity. However, under RTFOT and PAV conditions, the total functional group index of the SBS-modified asphalt is comparable to or higher than that of the base asphalt, reflecting the contribution of the polymer component itself to the measured indices. However, the absolute values of individual indices should be interpreted with caution due to possible interference from the polymer component. The relative difference of the I_S=O_ group index widened in the PAV stage, mainly due to the faster formation rate of the I_S=O_ group in the long-term aging of base asphalt. Consistent with the above reasoning, cross-type comparisons of individual I_C=O_ values are not attempted. Instead, the total functional group index (I_C=O_ and I_S=O_) is used to compare the overall oxidation degree between the two asphalt types. Under UV aging conditions, the SBS-modified asphalt exhibits a lower total functional group index than the base asphalt, indicating better overall anti-oxidative capacity during UV exposure.

## 4. Conclusions

In this study, the aging process of asphalt under construction, long-term service and light–temperature coupling was simulated by RTFOT, PAV and the equivalent radiation method. Macro- and micro-test methods, namely bending beam rheometer (BBR), atomic force microscope (AFM) and Fourier transform infrared spectroscopy (FTIR), were systematically evaluated for base asphalt and SBS-modified asphalt. The main conclusions were obtained as follows.

(1) The influence degree of three simulated aging methods on asphalt performance is ranked as follows: PAV is greater than RTFOT, approximately equal to 13 h UV. Long term thermal oxidative aging (PAV) has the most significant effect on the deterioration of low-temperature crack resistance, while short-term thermal oxidative aging (RTFOT) and 13 h ultraviolet aging have similar effects, indicating that, within the scope of the low-temperature rheological properties evaluated in this study and under the specific equivalence assumptions of the adopted accelerated protocol, the ultraviolet exposure corresponding to approximately one month of service in the high-altitude region does not induce obvious additional deterioration beyond that of RTFOT aging. This finding should not be generalized to other performance metrics or field conditions without further validation.
(2) UV aging promotes the formation of bee-like structures in base asphalt. Qualitatively, the number and area fraction of bee-like structures tended to increase initially and then decrease with increasing UV duration. For SBS-modified asphalt, the bee count remains lower than for base asphalt under equivalent conditions, indicating that the polymer network partially suppresses the colloidal restructuring caused by aging.
(3) There are significant differences in the macro- and micro-performance evolution of different modified asphalt during light–temperature coupling aging. The observed changes in I_C=O_ for SBS-modified asphalt after long-term aging may be associated with degradation of the polybutadiene phase. In the case of SBS-modified asphalt, I_S=O_ increases monotonically, whilst I_C=O_ exhibits a fluctuating pattern, initially increasing and then decreasing. The addition of SBS modifier effectively absorbs light fractions and forms a crosslinked network, thereby inhibiting the formation of oxygen-containing polar functional groups and enhancing the asphalt’s resistance to UV oxidation.

This study aims to compare the variations in SBS-modified asphalt with different aging methods; a more realistic natural aging process in northwestern China will be simulated using UV radiation alternating with periods of immersion in water.

## Figures and Tables

**Table 1:** ZH90# base asphalt technical indicators [23].

Indicators		Unit	Measured Values	Specification Requirements
Penetration (25 °C, 100 g, 5 s)		0.1 mm	87.2	80~100
Ductility (5 cm/min, 10 °C)		cm	>100	≥100
Softening point		°C	47.4	≥42
After RTFOT (85 min)	Penetration ratio	%	66	≥54
	Loss of mass	%	0.06	−0.8~0.8
	Ductility	cm	9.1	≥6

**Table 2:** Evaluation methods and indicators in this study.

Evaluation Methods	Evaluation Indicators	Symbols and Formulas
BBR Test	Creep stiffness modulus	S
	Creep rate	m-value
FTIR Analysis	Carbonyl index	$I_{C=O}=\frac{A_{C=O}}{A_{ref}}$
	Sulfoxide index	$I_{S=O}=\frac{A_{S=O}}{A_{ref}}$

## Data Availability

The original contributions presented in the study are included in the article. Further inquiries can be directed to the corresponding author.
